# Lumbar spine abnormalities in patients with obstructive sleep apnoea

**DOI:** 10.1038/s41598-021-95667-3

**Published:** 2021-08-10

**Authors:** Adam Domonkos Tarnoki, David Laszlo Tarnoki, Csaba Oláh, Marcell Szily, Daniel T. Kovacs, András Dienes, Marton Piroska, Bianka Forgo, Marina Pinheiro, Paulo Ferreira, László Kostyál, Martina Meszaros, Judit Pako, Laszlo Kunos, Andras Bikov

**Affiliations:** 1 Medical Imaging Centre, Semmelweis University, 78/A Üllői street, 1082 Budapest, Hungary; 2 Department of Neurosurgery, Borsod-Abaúj-Zemplén County and University Teaching Hospital, Miskolc, Hungary; 3 Department of Radiology, Faculty of Medicine and Health, Örebro University, Örebro, Sweden; 4 Faculty of Health Sciences, Discipline of Physiotherapy, The University of Sydney, Musculoskeletal Health, Sydney, Australia; 5 Department of Pulmonology, Semmelweis University, Budapest, Hungary; 6 Department of Pulmonology, University Hospital Zurich, Zurich, Switzerland; 7 National Koranyi Institute for Pulmonology, Budakeszi, Hungary; 8 Wythenshawe Hospital, Manchester University NHS Foundation Trust, Manchester, UK; 9 Division of Infection, Immunity and Respiratory Medicine, University of Manchester, Manchester, UK

**Keywords:** Diseases, Rheumatology, Comorbidities, Pain, Respiratory signs and symptoms, Neuroscience, Diseases of the nervous system

## Abstract

Previous studies suggested cervical spondylosis as a risk factor for development of obstructive sleep apnoea (OSA). We aimed to assess lumbar disc degeneration in patients with OSA and correlate the findings with symptoms and disease severity. Twenty-seven patients with OSA and 29 non-OSA controls underwent sleep studies and lumbar magnetic resonance imaging (MRI), and completed the Epworth Sleepiness Scale and the 24-item Roland‐Morris Disability Questionnaire (RMDQ) questionnaires. Plasma klotho was determined with enzyme-linked immunosorbent assay. Patients with OSA had higher number of disc bulges (4.6 ± 3.7 vs. 1.7 ± 2.5, p < 0.01) and anterior spondylophytes (2.7 ± 4.2 vs. 0.8 ± 2.1, p < 0.01), increased disc degeneration (total Pfirrmann score 16.7 ± 4.7 vs. 13.2 ± 4.1, p < 0.01) and vertebral fatty degeneration (7.8 ± 4.7 vs. 3.8 ± 3.7, p < 0.01). There was no difference in the RMDQ score (0/0–3.5/ vs. 0/0–1/, p > 0.05). Markers of OSA severity, including the oxygen desaturation index and percentage of total sleep time spent with saturation < 90% as well as plasma levels of klotho were correlated with the number of disc bulges and anterior spondylophytes (all p < 0.05). OSA is associated with lumbar spondylosis. Our study highlights the importance of lumbar imaging in patients with OSA reporting lower back pain.

## Introduction

Obstructive sleep apnoea (OSA) is a common disease which is characterised by recurrent episodes of partial or total collapse of the upper airways during sleep^1^. Chronic pain is a common symptom in patients with sleep disorders and people with chronic pain often have difficulties with their sleep^2^. If chronic pain, sleep disturbances, and mood disorders are present at the same time, an umbrella term of fibromyalgia is often used^3^. The link between sleep disorders and chronic pain is related to genetic and neuroendocrine factors, altered sensitivity of pain and systemic inflammation^2,4^. Magnetic resonance imaging may reveal structural abnormalities in these patients^5^.

Previous studies focusing on cervical spine reported that OSA was associated with increased number of cervical fusions and osteophytes^6^. More recently, cervical spondylosis was reported as a risk factor for OSA^7^. Yang et al. hypothesised that spondylosis leads to increased collapse potential of the upper airways, as the posterior pharyngeal wall is displaced anteriorly^7^. Interestingly, no studies assessed the lumbar spine region in OSA, despite the fact that around one third of patients with chronic back pain had abnormalities in their lumbar region^8^. The study of Yang suggested a unilateral causal relationship between cervical spondylosis and OSA. However, there is some evidence that OSA could also lead to spinal deformities directly through chronic intermittent hypoxia^9^ and indirectly through vitamin D deficiency^10^. Most importantly, intermittent hypoxia may lead to oxidative stress and inflammation in the lumbar discs which could contribute to spinal disc degeneration^11^. A potential molecule which could be a link between OSA and discopathy is the anti-inflammatory klotho protein, which levels were found decreased both in plasma samples of patients with OSA^12^ and in the nucleus pulposus specimens in disc degeneration^13^.

The prevalence rate of OSA in patients with chronic pain is 32%^14^, and the prevalence of excessively sleepy patients with OSA among those with chronic pain is 14%^15^. This is compatible with the estimated prevalence of OSA and excessively sleepy OSA in the general adult population^16^. In line with this, no association has been found with OSA and musculoskeletal pain^17^. However, it has been demonstrated that chronic pain may impair normal sleep architecture^14^ and has significant consequences on the quality of life in patients with OSA^17^.

We hypothesised that OSA may lead to disc degeneration which could eventually contribute to chronic pain. To check on this, we performed a sleep study, a lumbar MRI and measured plasma klotho levels in patients with OSA and non-OSA controls.

## Methods

### Study subjects and design

All subjects from the Hungarian Twin Registry^18^ who attended a sleep study^19^ were invited to have a lumbar magnetic resonance imaging (MRI). None of the subjects had previously been diagnosed with obstructive sleep apnoea or lumbar spinal disease, hence none received any treatment for these. Exclusion criteria included acute heart failure, pregnancy, breastfeeding, those with a positive pregnancy test, immunosuppressive/immunomodulatory therapy in the last 30 days, including systemic steroid-containing medicines, chemotherapy in the last year, major surgery in the last 2 months, transfusion, receiving other blood products in the last 2 months, pacemaker, ICD, other implanted device, magnetisable metal object in the body, claustrophobia and aphasia. Comorbidities were derived from subject reports and their medications. If both members of the twin pair were eligible, only one of them was randomly selected using an online platform (https://www.sealedenvelope.com/simple-randomiser/v1/lists).

The subjects first attended the Sleep Laboratory, Department of Pulmonology, Semmelweis University. In the evening after taking their medical history, they filled out the Epworth Sleepiness Scale (ESS), the 24-item Roland‐Morris Disability Questionnaire (RMDQ) and a custom made back-pain questionnaire (Supplement 1). This was followed by an inpatient polysomnography (n = 39) or cardiorespiratory polygraphy (n = 17). The type of the sleep test was decided based on the pre-test probability of OSA. Patients with higher likelihood for OSA (i.e. snoring, obesity) had cardiorespiratory polygraphy. The following day fasting blood was taken for klotho which was measured with commercially available enzyme-linked immunosorbent assay as described previously^12^.

Patients then attended a lumbar spine MRI on a Siemens Magnetom Verio 3T scanner in the Borsod-Abaúj-Zemplén County Hospital (Miskolc, Hungary) or on a Philips Ingenia 1.5T in the Medical Imaging Centre of Semmelweis University (Budapest, Hungary).

This study was conducted in accordance with the Declaration of Helsinki and approved by the local Ethics Committees (Semmelweis University TUKEB 30/2014 and Borsod-Abaúj-Zemplén County Hospital in Miskolc, Hungary). All volunteers gave their written informed consent before participating in the study. The study was not preregistered.

### Sleep studies

Sleep studies were performed with the Somnoscreen Plus Tele PSG and the Somnoscreen RC devices (Somnomedics GmbH) between 22:00 and 06:00 as described previously^20^. Apnoea was defined by a 90% decrease of the nasal airflow, which lasted for more than 10 s, and hypopnea was defined as at least 30% airflow decrease lasting for at least 10 s, which related to a ≥ 3% oxygen desaturation or an arousal. We recorded the total sleep time (TST) and sleep period time (SPT). Apnoea-hypopnoea index (AHI), oxygen desaturation index (ODI) and percentage of time spent with oxygen saturation below 90% (TST90%) were calculated as markers of OSA severity. Sleep efficiency was calculated as TST/SPT. OSA was defined by an AHI ≥ 5/hours.

### Lumbar magnetic resonance imaging

Sagittal T1-weighted, T2-weighted, short tau inversion recovery (STIR) and axial T2-weighted images were performed. Grading was performed on T2-weighted and STIR images retrospectively by two experienced radiologists. Axial sections were obtained at selected levels to assess structural changes in individuals who had features suggesting prolapse.

The presence or absence of disc dehydration, disc height narrowing, disc bulging and disc herniation was noted based on the standardized criteria^21–23^. Disc dehydration was established based on sagittal T2-weighted images when signal intensity of the disc was lower compared to the cerebrospinal fluid. We did not distinguish between the varying levels of dehydration. Disc height narrowing was registered when the height of the disc proved to be equal or lower than the one directly cranial to it on sagittal T1-weighted images. Disc height narrowing of the L5 disc was registered only if its height was lower than that of the L4 disc. In case of an intervertebral disc material extending beyond the space between the vertebral bodies, bulging or herniation was noted when the overextension was broad (more than 25% of disc circumference) or narrow (less than 25% of disc circumference) respectively on axial T2-weighted images. All disc bulging and hernia were counted and summarized as a total value between Th12 and S1 segments. Total endplate score (TEPS) was used to assess the level of endplate degeneration^24^. Endplate defect was evaluated on T1-weighted scans and classified into six types according to severity of damage (type 1 to type 6) assessed on T1-weighted sequences. TEPS was derived from each disc by adding up the endplate defect scores of both rostral and caudal endplates of the disc. Disc degeneration was assessed by the Pfirrmann’s grading (Pfirrmann score) on T2-weighted images and discs were considered to be healthy if they were Grade I, II or III and degenerated if they were Grade IV or V^25^ and the total score for the entire lumbar spine was calculated which was a sum of each disc’s Pfirrmann score. Due to the degeneration of the disc and small joints, the vertebrae may move. In order to characterize this feature of degeneration, the presence and severity (length compared to the lower vertebral body) of anterolisthesis, which refers to anterior displacement (forward slip) of a vertebral body relative to the one below, and retrolisthesis was also recorded^26^. To evaluate superior and inferior endplate disease, each endplate was evaluated on T1-weighted scans and classified into 4 types as no abnormality (0), mild (1 to 5 mm), moderate (5 to 10 mm) and severe (over 10 mm) defect and the average score was calculated for the entire lumbar spine. Presence (1) or absence (0) of superior and inferior Schmorl nodes, defined as endplate breaks with depression of subchondral bone of incremental severity, were recorded on T1- or T2- weighted scans. Vertebral fatty degeneration was analysed on T1-weighted scans and characterized as none (0), mild (1, up to 25% fatty infiltration), moderate (2, 25–50% fatty infiltration) and severe (3, 50–100% fatty infiltration) and total score for the lumbar spine was calculated. The presence of anterior and posterior spondylophytes was recorded. The presence of annular high intensity zone (HIZ), a high-intensity focal signal on T2-weighted sequences in the posterior annulus fibrosus with a considerably brighter signal intensity than nucleus pulposus that is distinctly dissociated, was marked^27^.

### Statistical analysis

The Statistica 12 (StatSoft, Inc., Tulsa, OK, US) software was used for analysis. The normality of the data was assessed with the Kolmogorov–Smirnov test. The OSA and control groups were compared with t-test, Mann–Whitney, Fisher and Chi-square tests. Comparisons of back pain and MRI results were adjusted for age, gender, body mass index (BMI), smoking history, cardiovascular and cerebrovascular diseases and the type of the sleep test. Sleep parameters were correlated with outcomes of pain questionnaires and MRI parameters using Spearman’s correlation (continuous variables), negative binomial model (count variables) and logistic regression (dichotomous variables). The latter two analyses were adjusted for gender, age, BMI, cardiovascular disease, smoking and the type of the sleep test. The relationship between discopathy and chronic pain was assessed with Spearman’s correlation, logistic regression analysis or negative binomial model following adjustment for age, gender, BMI and ODI. The relationship between klotho levels lumbar spine pathology was assessed with Spearman test, logistic regression or negative binomial model adjusted for gender, age, BMI and ODI. The analyses were performed after excluding patients with osteoporosis and those taking painkillers (n = 13). A p value < 0.05 was considered significant. Data are presented as mean ± standard deviation or median/interquartile range/.

The primary aim of the study was to analyse the relationship between OSA and lumbar spondylosis. As the results were obtained from a limited population, no formal power analysis was performed. Post-hoc sensitivity analyses showed that we were able to detect an effect size greater than 0.67 with a power of 0.80 and an alpha error of 0.05.

## Results

### Comparison of the OSA and control groups

OSA was diagnosed in 27 participants, 21 had mild (AHI < 15/h) disease. Patients with OSA were older, had higher prevalence of smoking history, hypertension, cardiovascular or cerebrovascular disease and osteoporosis (all p < 0.05) and tended to have higher BMI (p = 0.05). As expected, AHI, ODI and TST90% was higher in patients with OSA (p < 0.05, Table 1).

**Table 1:** Comparison of demographics and clinical characteristics of patients with OSA and controls.

	OSA (n = 27)	Controls (n = 29)	P
Age (years)	60/53–68/	41/28–54/	< 0.01
Gender (male %)	33	28	0.64
BMI (kg/m^2^)	26.6/23.3–29.6/	24.4/21.0–27.7/	0.05
Smoker (ever %)	26	3	0.01
Hypertension (%)	67	28	< 0.01
Cardiovascular or cerebrovascular disease (%)	26	3	0.01
Depression (%)	7	3	0.51
Diabetes (%)	22	10	0.22
Dyslipidaemia (%)	48	38	0.44
Asthma (%)	11	7	0.58
COPD (%)	7	3	0.51
Osteoporosis (%)	19	0	0.01
Aspirin use	7	22	0.03
Opioid use	4	0	0.30
ESS	6/4.5–10.5/	6	0.76
TST (min)	394/378–430/	416/362–424/	0.67
SPT (min)	435/406–462/	425/407–437/	0.41
Sleep %	95/89–98/	96/92–99/	0.32
AHI (1/h)	9.4/6.5–14.2/	2.3/1.0–2.8/	< 0.01
ODI (1/h)	8.4/4.9–11.2/	0.9/0.2–1.7/	< 0.01
TST90% (%)	1.3/0.2–2.9/	0.0/0.0–0.0/	< 0.01

*AHI* apnoea-hypopnoea index, *BMI* body mass index, *COPD* chronic obstructive pulmonary disease, *ESS* Epworth Sleepiness Scale, *ODI* oxygen desaturation index, *OSA* obstructive sleep apnoea, Sleep%—sleep efficiency, *SPT* sleep period time, *TST* total sleep time, *TST90%* percentage of total sleep time spent with oxygen saturation below 90%. Data are presented as median /interquartile range/

### Lumbar spine spondylosis in patients with OSA and controls

Patients with OSA had an increased number of disc bulges (p < 0.01, after exclusion p < 0.01) and a higher Pfirrmann score (p < 0.01, after exclusion p = 0.05). We detected increased number of anterior spondylophytes (p = 0.01) and increased magnitude of vertebral fatty degeneration (p < 0.01) in patients with OSA compared to the controls. However, after excluding patients with osteoporosis and those who were taking painkiller, these differences became insignificant (p = 0.07 for the number of anterior spondylophytes, p = 0.06 for the magnitude of vertebral fatty degeneration). There was no difference in any other degenerative parameter (Table 2).

**Table 2:** Characteristics of lumbar spine spondylosis and discopathy in patients with OSA and controls.

	OSA (n = 27)	Controls (n = 29)	P
Disc hernia (at least one, %)	28	26	0.92
Number of all disc hernias (n)	0.6 ± 1.0	0.3 ± 0.5	0.92
Total endplate score (n)	20.9 ± 7.8	20.8 ± 9.7	0.38
Disc bulging (at least one, %)	85	52	< 0.01
Number of all disc bulgings (n)	4.6 ± 3.7	1.7 ± 2.5	< 0.01
Total Pfirrmann score (n)	16.7 ± 4.7	13.2 ± 4.1	< 0.01
Anterolisthesis (at least one, %)	15	3	0.13
Anterolisthesis (mm)	0.7 ± 1.8	0.1 ± 0.7	0.13
Retrolisthesis (at least one, %)	12	3	0.28
Retrolisthesis (mm)	0.3 ± 1.0	0.2 ± 1.1	0.28
Superior endplate disease (n)	0.8 ± 1.6	0.7 ± 2.1	0.15
Inferior endplate disease (n)	0.8 ± 1.3	0.7 ± 2.0	0.44
Superior Schmorl nodes (n)	0.8 ± 1.2	0.9 ± 1.4	0.90
Inferior Schmorl nodes (n)	0.4 ± 0.8	0.7 ± 1.1	0.56
Vertebral fatty degeneration (score between 0 and 18)	7.8 ± 4.7	3.8 ± 3.7	< 0.01
Anterior spondylophytes (n)	2.7 ± 4.2	0.8 ± 2.1	0.01
Posterior spondylophytes (n)	1.1 ± 2.4	0.4 ± 0.9	0.47
Disc height narrowing (mm)	3.3 ± 2.1	3.0 ± 3.2	0.15
Annular high intensity zone (n)	0.6 ± 1.2	0.3 ± 0.6	0.40

Analyses were adjusted for age, gender, BMI, smoking and cardiovascular disease. Data are presented as mean ± standard deviation.

**Figure 1:**
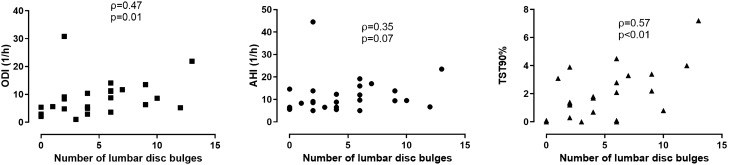
The relationship between the number of lumbar disc bulges and markers of OSA severity.

**Figure 2:**
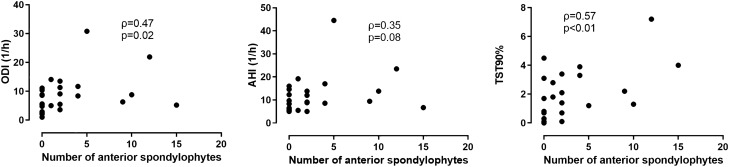
The relationship between the number of anterior vertebral spondylophytes and markers of OSA severity.

Analysing all subjects together, there were significant correlations between the number of disc bulges and markers of OSA severity, such as AHI, ODI, TST90% (all p < 0.05) as well as the number of anterior spondylophytes and AHI, ODI and TST90% (all p < 0.05). These associations were significant (all p < 0.05) even when the 13 subjects were excluded except for the relationship between AHI and anterior spondylophytes (p = 0.07). When only patients with OSA were analysed, significant relationships were present only with markers of overnight hypoxaemia (Figs. 1 and 2). Of note, a direct relationship was noted between the number of disc bulges and the number of anterior spondylophytes (ρ = 0.54, p < 0.01, after exclusion ρ = 0.51, p < 0.01). The relationship was present even following further adjustment for the presence of OSA.

The number of posterior spondylophytes correlated only with ODI (ρ = 0.32, p = 0.01, after exclusion ρ = 0.38, p = 0.01). The Pfirrmann score significantly related to AHI (ρ = 0.40, p < 0.01, after exclusion ρ = 0.34, p = 0.03), ODI (ρ = 0.56, p < 0.01, after exclusion ρ = 0.49, p < 0.01) and TST90% (ρ = 0.61, p < 0.01, after exclusion ρ = 0.59, p < 0.01). There was a significant relationship between the disc height narrowing and ODI (ρ = 0.28, p < 0.05). However, after excluding patients with osteoporosis and those who were taking painkillers, this became insignificant (ρ = 0.21, p = 0.18). Disc height narrowing significantly correlated with TST90% (ρ = 0.29, p < 0.05, after exclusion ρ = 0.31, p = 0.05). None of the other sleep parameters or ESS correlated with any characteristics of lumbar spondylosis (all p > 0.05).

### Chronic pain in patients with OSA and controls

There was no difference in the life-time prevalence of lower back pain between the OSA (85%) and control groups (72%, p = 0.78, after exclusion p = 0.85). Thirty-eight percent of the patients and 34% of controls reported lower back pain in the last 4 weeks (p = 0.41, after exclusion p = 0.77). Fifty-five percent of the patients and 28% controls reported that lower back pain limits their daily activities (p = 0.56, after exclusion p = 0.32). However, there was no difference in the RMDQ score between the two groups (0/0–3.5/ vs. 0/0–1/; OSA vs. controls, respectively, p = 0.29, after exclusion p = 0.25). Similarly, no significant difference was found regarding responses to other questions between the two groups (all p > 0.05).

None of the sleep parameters related to the life prevalence of lower back pain or lower back pain in the last 4 weeks (p > 0.05, after exclusion p > 0.05). There was a tendency for a direct relationship between RMDQ and ESS (ρ = 0.27, p = 0.07). After excluding the patients with osteoporosis, and those who were taking painkillers, this relationship became significant (ρ = 0.38, p = 0.03). There was no relationship between chronic pain and BMI (p > 0.05, after exclusion p > 0.05).

### The relationship between lumbar spine spondylosis, discopathy and chronic pain

**Figure 3:**
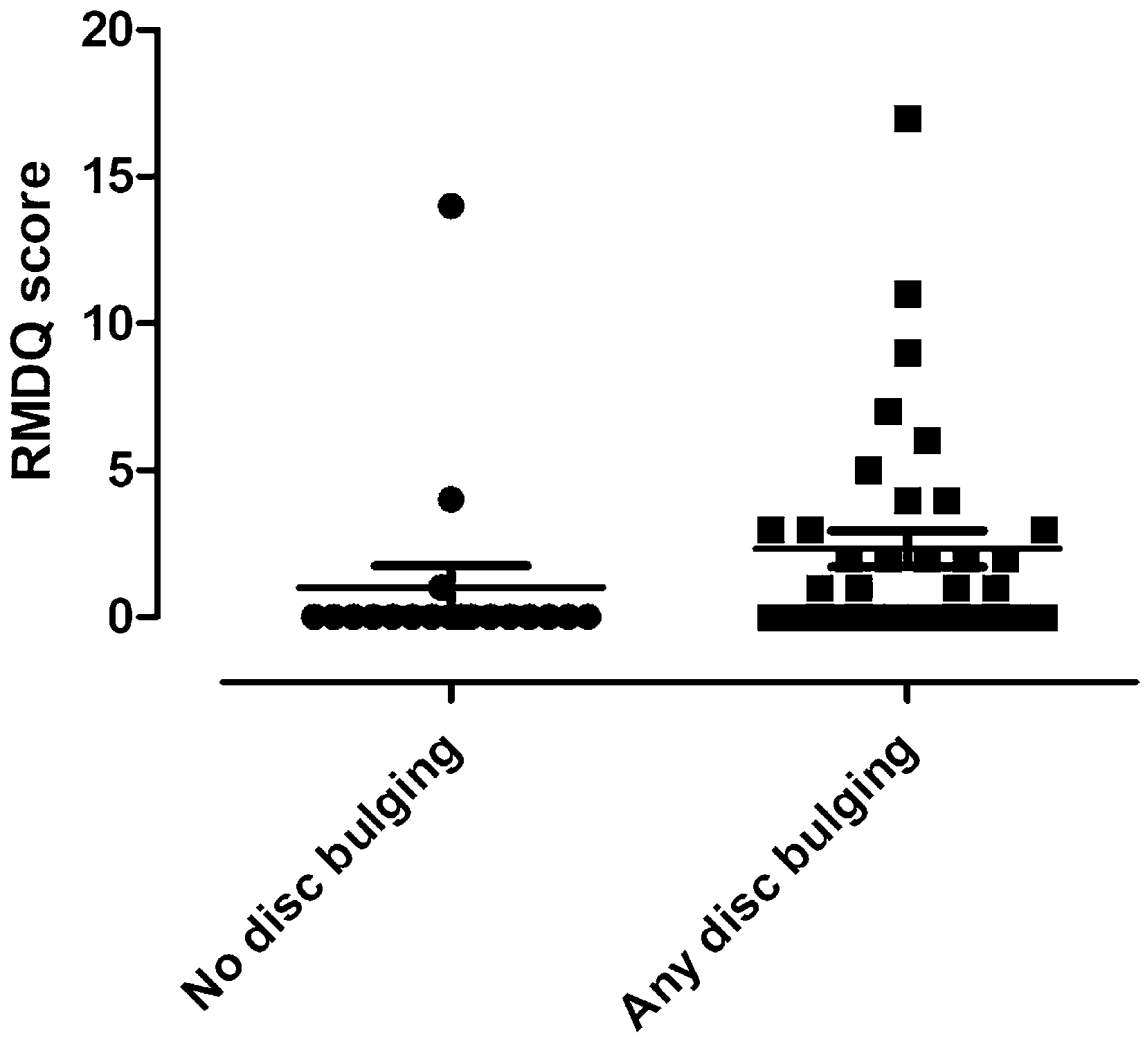
The Roland‐Morris Disability Questionnaire score in subjects with and without lumbar disc bulges.

Participants who had any disc bulging had a higher RMDQ score (1/0–3/ vs. 0/0–0/, p = 0.01, Fig. 3) even following excluding the 13 subjects (p = 0.03). Interestingly, there was no relationship between the number of disc bulges and RMDQ (p = 0.20) or the other pain outcomes (p > 0.05).

There was a significant relationship between the Pfirrmann and the RMDQ score (ρ = 0.32, p = 0.02). After excluding the patients mentioned above, this relationship became insignificant (ρ = 0.09, p = 0.56). There was also a significant relationship between the RMDQ score and number of superior endplate disease, inferior endplate disease as well as the number of anterior spondylophytes (all p < 0.05). After excluding patients, only the relationship between RMDQ and the number of anterior spondylophytes remained significant (ρ = 0.46, p < 0.01). None of the other characteristics of spondylosis or discopathy were related to measures of chronic pain.

### The role of klotho in lumbar spine spondylosis and discopathy associated with OSA

**Figure 4:**
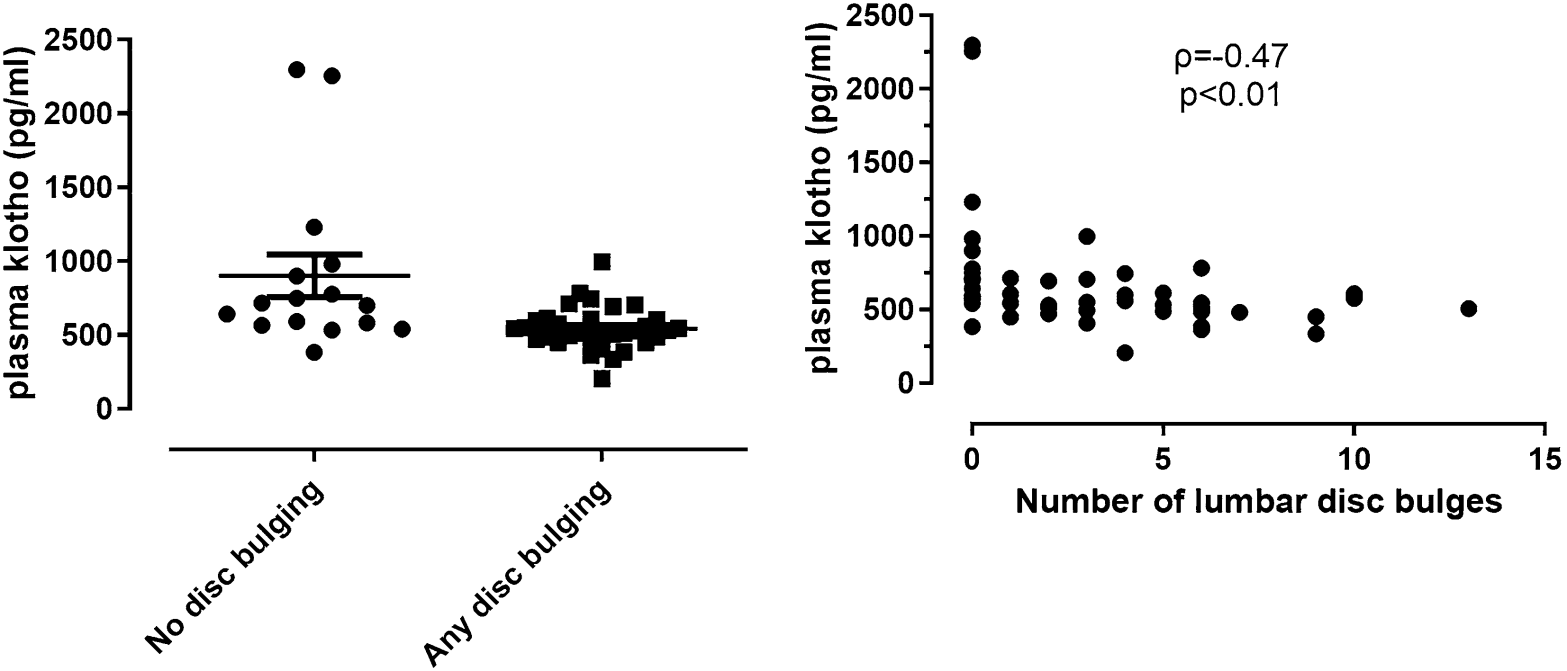
The relationship between plasma klotho levels and lumbar disc bulges.

Plasma klotho levels were inversely related to the number of disc bulges (ρ = − 0.47, p < 0.01, after exclusion ρ = − 0.49, p < 0.01, Fig. 4), and there was a tendency for an inverse correlation with total endplate score (ρ = − 0.26, p = 0.07) as well as annular high intensity zones (ρ = − 0.27, p = 0.06). These remained insignificant after excluding the patients mentioned above (all p > 0.05). However, there was no further relationship with any other characteristics of spondylosis or discopathy (all p > 0.05).

There was no relationship between plasma klotho and BMI (p = 0.33) or age (p = 0.09), whilst it significantly related to AHI (ρ = − 0.33, p = 0.03), ODI (ρ = − 0.33, p = 0.02) and TST90% (ρ = − 0.44, p < 0.01). Although klotho levels did not differ between the OSA and control groups (p = 0.15), patients OSA with at least one anterior spondylophyte had significantly lower klotho levels compared to the controls with at least one anterior spondylophyte (p < 0.01). However, there was no difference in klotho levels between the two groups in those patients who had at least one disc bulging (p = 0.93).

## Discussion

According to our knowledge, this is the first study investigating the relationship between obstructive sleep apnoea and lumbar spine abnormalities. We found that OSA was significantly associated with the presence of certain abnormalities, most strongly with disc bulges and anterior spondylophytes.

Previous studies focusing on the cervical spinal region reported increased number of spondylophytes in OSA^7,28^ and suggested they may contribute to the development of sleep apnoea^7^. Spondylophytes are bony projections that form along joint margins. Usually, they are associated with the degeneration of the lumbar disc which starts losing water content and leads to a decrease in the intervertebral disc height. To compensate the loss of the flexibility of the annulus fibrosus, apophyseal joints start to grow a new bone^6,28^. In line with this, we found a significant direct relationship between the number of spondylophytes and disc bulges. However, the design of the study cannot answer the causality of the relationship as both abnormalities may have been driven by OSA, independently. Our study extends the previous observations on the cervical spine region to the lumbar region, as the number of disc bulges and spondylophytes were higher in OSA and related to disease severity.

The relationship between OSA and disc degeneration can be explained by multiple mechanisms. Several genes of collagen biosynthesis and inflammation were found to be associated with OSA^29^ and disc degeneration^30^ as well suggesting for a common genetic origin. Age, male gender and obesity, risk factors for OSA^1^, are associated with an increased likelihood for the development of disc degeneration^30,31^. Of note, the relationship between OSA and discopathy in the present study was present after adjustment for these factors. OSA is also associated with abnormal bone formation likely due to the accelerated remodelling induced by intermittent hypoxia^9^ and vitamin D deficiency in sleep apnoea^10^. Chronic intermittent hypoxia in OSA leads to oxidative stress and inflammation^1^, both of which are associated with disc degeneration^30^. More particularly, reactive oxygen species can induce the expression of extracellular matrix proteases and proinflammatory markers in disc cells^32^. Supporting this, a direct relationship was seen between markers of overnight hypoxaemia and markers of disc degeneration in the current study. In addition, systemic inflammation generated in OSA may also contribute to inflammation in lumbar disc^33^.

A potential molecule which is involved in the pathomechanism of both diseases is the klotho protein^12,13^. This protein is synthesised predominantly in the kidneys, but other organs, such as parathyroid gland, ovary, testis and placenta also express the klotho gene^34^. Klotho is involved in the phosphate homeostasis, it regulates the aldosterone synthesis, has an anti-aging, anti-oxidative stress and anti-inflammatory role^35^. We have reported low levels of klotho in OSA which were associated with markers of overnight hypoxaemia^12^. Hypoxaemia^36^ and oxidative stress^13^ lead to decreased klotho expression in nucleus pulposus cells. Klotho attenuates inflammation at the same site^13^ and it is hypothesised that lower klotho levels contribute to disc degeneration^13^. Furthermore, klotho gene polymorphisms were associated with lumbar spondylosis^37^.

In line with previous reports^17^, we did not find a significant difference in the reported back pain between patients with OSA and controls. In contrast, back pain was associated with increased daytime sleepiness. Chronic pain can disturb the normal sleep architecture^14^ with subsequent daytime effects. Although excessive daytime sleepiness is a common symptom of OSA^1^, our study highlights that other factors than OSA can lead to this symptom and these should be explored in clinical practice. Most particularly, clinicians should not accept chronic pain as a natural component of the fibromyalgia syndrome but may investigate anatomic causes for pain and sleepiness. In contrast to OSA, chronic pain was associated with lumbar spondylosis and discopathy. This may highlight an indirect mechanism between spondylosis and OSA development, as chronic pain may limit mobility eventually leading to weight gain. Obesity is a leading cause for OSA and limited exercise could subsequently cause OSA development^1^. A previous meta-analysis concluded that obesity is significantly associated with low back pain^38^. However, our study did not find any relationship between BMI and pain outcomes. Notably, due to the subjective nature of the outcome and the limited number of subjects, the results need to be interpreted carefully.

Our study has limitations. First, the number of subjects was relatively low, and increasing the numbers more complex relationships could be detected. Second, two different MR machines were used. Third, most of the patients with OSA had mild disease. Enrolling patients with more severe disease could reveal more precisely the relationship between OSA and lumbar spine degeneration. More importantly, the lack of relationship between OSA and chronic pain could be due to the milder disease severity. Of note, the relationship between OSA and disc bulges as well as spondylophytes was evident even at this severity. Fourth, some patients had cardiorespiratory polygraphy instead of polysomnography. Polygraphy may underestimate the severity of OSA, as hypopnoeas associated with arousals but without desaturations are not counted and the total number of respiratory events is divided by the total time recorded rather than the total sleep time. Acknowledging this bias, the results were adjusted for the type of the sleep test. Fifth, the groups were not balanced in terms of age, gender, BMI and comorbidities. Although the analyses were adjusted for these factor, further studies in larger populations are warranted to minimise this bias. Sixth, physical activity may influence the results, however this has not been recorded in this study. Finally, none of the patients were treated with continuous positive airway therapy (CPAP). Follow up trials are warranted to see if CPAP could be beneficial in preventing or slowing down spondylosis in patients with OSA. Interventional trials may also reveal independent associations between OSA and spondylosis not biased by confounders. Despite these limitations, we believe that our pioneering study may serve a potential basis for designing large scale studies.

In summary, we report that markers of OSA severity is associated with lumbar spondylosis. Our study highlights the importance of lumbar imaging in patients with OSA reporting lower back pain.

## Supplementary Information

Supplementary Information.

## Data Availability

The datasets generated during and/or analysed during the current study are available from the corresponding author on reasonable request.
