# Supplementary material for: Lumbar spine abnormalities in patients with obstructive sleep apnoea
Source: Sci Rep. 2021 Aug 10;11:16233. doi: 10.1038/s41598-021-95667-3 (PMC8355280; doi:10.1038/s41598-021-95667-3)
Supplement: Supplementary file 1 — Supplementary Information. [file 41598_2021_95667_MOESM1_ESM.docx]

**Lumbar spine abnormalities in patients with obstructive sleep apnoea**

Adam Domonkos Tarnoki, David Laszlo Tarnoki, Csaba Oláh, Marcell Szily, Daniel T Kovacs, András Dienes, Marton Piroska, Bianka Forgo, Marina Pinheiro, Paulo Ferreira, László Kostyál, Martina Meszaros, Judit Pako, Laszlo Kunos, Andras Bikov

**Supplement 1.**

**Questionnaire about lower back pain**

a.1. Have you ever had lower back pain?

Yes 🞏 No 🞏

a.2. Does lower back pain limit your normal daily activities or change your daily routine more than one day?

Yes 🞏 No 🞏

a.3. When was the last time when you had lower back pain?

In the last 2 years 🞏 Last year 🞏 In the last 6 months 🞏 In the last 4 weeks 🞏

a.4. Did you have lower back pain in the last 4 weeks? (Except for lower back pain due to infection with high temperature or menstruation)

Yes 🞏 No 🞏

a.5. If yes, did this lower back pain limit your normal daily activities or change your daily routine more than one day?

Yes 🞏 No 🞏

a.6. How long did this pain take?

Less than 3 months 🞏 More than 3 months, but less than 7 months 🞏

7 months or more, but less than 3 years 🞏 3 years or more 🞏

a.7. Please sign (with X in the table below), how was the intensity of your pain on a 0-10 scale, where 0 means “No pain” and 10 means “Worst possible pain”:

… the intensity of the last lower back pain:

| 0 | 1 | 2 | 3 | 4 | 5 | 6 | 7 | 8 | 9 | 10 |
| --- | --- | --- | --- | --- | --- | --- | --- | --- | --- | --- |
| No pain |  |  |  |  |  |  |  |  |  | Worst possible pain |

… the intensity of the worst lower back pain:

| 0 | 1 | 2 | 3 | 4 | 5 | 6 | 7 | 8 | 9 | 10 |
| --- | --- | --- | --- | --- | --- | --- | --- | --- | --- | --- |
| No pain |  |  |  |  |  |  |  |  |  | Worst possible pain |

a.8. If you had lower back pain in the last 4 weeks, how often did you have this pain?

Just some days 🞏 Most of the days 🞏 Everyday 🞏

a.9. How many different lower back pain episodes did you have in your life that were strong enough to pain limit your normal daily activities or change your daily routine more than one day? *(Different episode means that at least a month passed between the two episodes when you had no pain.)*

*How many times? _________________*

a.10. Were you unable to work or did you reduce your normal daily activities due to lower back pain in the last 12 months?

Yes 🞏 No 🞏

a.11. If yes, please estimate the total number of days when you dropped out of your job:

0-30 days **□** 30-60 days **□** 60-90 days **□** More than 90 days **□**
